# The complete mitogenome of sea hares, *Dolabella auricularia* (Mollusca: Aplysiidae)

**DOI:** 10.1080/23802359.2017.1365656

**Published:** 2017-08-17

**Authors:** Geng-Ming Lin, Peng Xiang, Gilbert Audira, Chung-Der Hsiao

**Affiliations:** a Laboratory of Marine Biology and Ecology, Third Institute of Oceanography, SOA, Xiamen, China;; b Department of Bioscience Technology, Chung Yuan Christian University, Chung-Li, Taiwan;; c Center for Biomedical Technology, Chung Yuan Christian University, Chung-Li, Taiwan;; d Center for Nanotechnology, Chung Yuan Christian University, Chung-Li, Taiwan

**Keywords:** Sea hares, *Dolabella auricularia*, mitogenome, next generation sequencing

## Abstract

In this study, the complete mitogenome sequence of sea hares, *Dolabella auricularia* (Mollusca: Aplysiidae), has been decoded for the first time by low coverage whole genome sequencing method. The overall base composition of *D. auricularia* mitogenome is 31.5% for A, 14.0% for C, 16.4% for G, and 38.0% for T, and has low GC content of 30.5%. The assembled mitogenome, consisting of 14,598 bp, has unique 13 protein-coding genes (PCGs), 22 transfer RNAs, and two ribosomal RNAs genes. The *D. auricularia* has the common mitogenome gene organization and feature of Aplysiidae. The complete mitogenome of *D. auricularia* provides essential and important DNA molecular data for further phylogenetic and evolutionary analysis for Aplysiidae.

Sea hares (Anaspidea) lives on the surface of the sea floor and its order includes 10 genera, including *Dalabella*. Most sea hares feed on a wide array of red and green algae. They are also known as voracious animals that devote certain hours a day to feeding and can consume a quantity of food reaching on third of their body weight every day (Heller 2015). This mollusc is also prominent to be a rich source of anticancer and/or cytostatic peptides such as dolastatins 10 and 15 that are currently under development by the NCI (Ishiwata et al. 1994). Molluscs comprise a highly diverse group of organisms and multiple taxa for which relationships can be difficult to determine due to the relatively high mutation rate of their mitogenomes and a remarkable amount of variation in gene rearrangement as compared to another species. In this research, we sequenced and characterized the complete mitogenome of *Dolabella auricularia* in an attempt to expand general knowledge of molluscan mitogenomes (Knudsen et al. 2006).

Samples (voucher no. 631) of *D. auricularia* were collected from local aquarium in Taiwan and originally imported from Indonesia. The methods for genomic DNA extraction, library construction, and next generation sequencing were described in our previous publication (Shen et al. 2016). Initially, the raw next generation sequencing reads generated from HiSeq X Ten (Illumina, San Diego, CA) were filtered to remove low quality reads. Around 0.04% raw reads (7386 out of 17,135,724) were subjected to *de novo* assembly using commercial software (Geneious V9, Auckland, New Zealand) to produce a single, circular form of complete mitogenome with about an average 79× coverage. The complete mitogenome of *D. auricularia* contains 14,099 bp in size (GenBank MF591574) with overall base composition of 28.3% for A, 15.4% for C, 19.6% for G, and 36.7% for T, and has low GC content of 35.0%, showing 77% identities to the complete mitogenome of *Aplysia kurodai* (GenBank KJ415053). The protein coding, rRNA, and tRNA genes of *D. auricularia* mitogenome were predicted by using DOGMA (Wyman et al. 2004), ARWEN (Laslett and Canback 2008), and MITOS (Bernt et al. 2013) tools, and manually inspected. The complete mitogenome of *D. auricularia* includes unique 13 protein-coding genes (PCGs), 22 transfer RNA genes, and two ribosomal RNA genes. The *D. auricularia* mitogenome has the common gene organization and feature with other Aplysiidae species. The longest gene of all PCGs is COX1 gene (1602 bp), whereas the shortest one is ATP8 gene (153 bp). The size of small ribosomal RNA (12S rRNA) and large ribosomal RNA (16S rRNA) genes is 725 bp and 1040 bp, respectively. The size of all tRNA ranged from 54 to 67 bp. Four PCGs (ATP8, ND3, ATP6, and COX3), one rRNA (12S rRNA), and eight tRNA (tRNA-Q, tRNA-L2, tRNA-N, tRNA-R, tRNA-E, tRNA-M, tRNA-S2, and tRNA-T) genes are encoded on reversed-strand, while other genes are encoded in the forward-strand.

To validate the phylogenetic position of *D. auricularia,* we used MEGA6 software (Tamura et al. 2013) to construct a maximum likelihood tree (with 500 bootstrap replicates) containing complete mitogenomes of nine species derived from Nudibranchia. *Hypselodoris festiva* derived from Chromodorididae was used as outgroup for tree rooting. Result shows *D. auricularia* can be unambiguously grouped with other Aplysiidae species having high bootstrap value supported (Figure 1). In conclusion, the complete mitogenome of the *D. auricularia* deduced in this study provides essential and important DNA molecular data for further phylogenetic and evolutionary analysis for Aplysiidae phylogeny.

**Figure 1.**
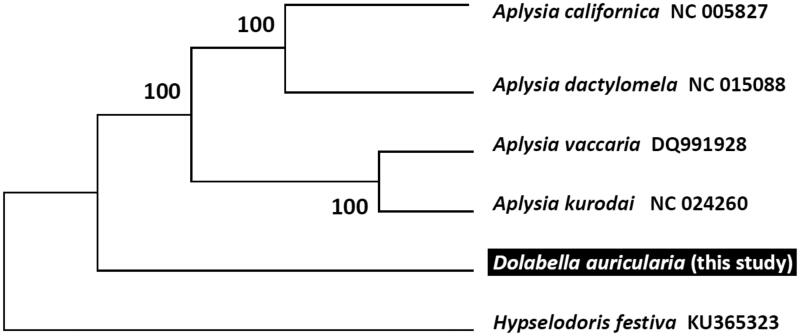
Molecular phylogeny of *Dolabella auricularia* and related species in Nudipleura based on complete mitogenome. The complete mitogenome is downloaded from GenBank and the phylogenetic tree is constructed by maximum likelihood method with 500 bootstrap replicates. The gene's accession number for tree construction is listed behind species name.
